# Peroxisome proliferator-activated receptor-γ agonist inhibits the mammalian target of rapamycin signaling pathway and has a protective effect in a rat model of status epilepticus

**DOI:** 10.3892/mmr.2015.3641

**Published:** 2015-04-17

**Authors:** YONG-ZHI SAN, YU LIU, YU ZHANG, PING-PING SHI, YU-LAN ZHU

**Affiliations:** Department of Neurology, Second Affiliated Hospital of Harbin Medical University, Harbin, Heilongjiang 150086, P.R. China

**Keywords:** epilepsy, epileptogenesis, mammalian target of rapamycin signaling pathway, peroxisome proliferator-activated receptor-γ

## Abstract

Peroxisome proliferator-activated receptor γ (PPAR-γ) has a protective role in several neurological diseases. The present study investigated the effect of the PPAR-γ agonist, pioglitazone, on the mammalian target of rapamycin (mTOR) signaling pathway in a rat model of pentylenetetrazol (PTZ)-induced status epilepticus (SE). The investigation proceeded in two stages. First, the course of activation of the mTOR signaling pathway in PTZ-induced SE was examined to determine the time-point of peak activity, as reflected by phopshorylated (p)-mTOR/mTOR and p-S6/S6 ratios. Subsequently, pioglitazone was administrated intragastrically to investigate its effect on the mTOR signaling pathway, through western blot and immunochemical analyses. The levels of the interleukin (IL)-1β and IL-6 inflammatory cytokines were detected using ELISA, and neuronal loss was observed via Nissl staining. In the first stage of experimentation, the mTOR signaling pathway was activated, and the p-mTOR/mTOR and p-S6/S6 ratios peaked on the third day. Compared with the vehicle treated-SE group, pretreatment with pioglitazone was associated with the loss of fewer neurons, lower levels of IL-1β and IL-6, and inhibition of the activation of the mTOR signaling pathway. Therefore, the mTOR signaling pathway was activated in the PTZ-induced SE rat model, and the PPAR-γ agonist, pioglitazone, had a neuroprotective effect, by inhibiting activation of the mTOR pathway and preventing the increase in the levels of IL-1β and IL-6.

## Introduction

Epilepsy is a common neurological disease, characterized by unpredictable recurrent seizures. The disease affects all age groups and, in the majority of cases, the cause is unknown. Susceptibility to epilepsy may be genetic, however, environmental factors, including stroke, brain cancer, brain trauma, and drug and alcohol misuse are also risk factors (1). Seizures are the direct result of the excessive and abnormal discharge of neurons (2). The majority of antiepileptic drugs work by interfering with ion channel function, to reduce or inhibit excitatory neurophysiological activity. However, they rarely affect epileptogenesis (3).

Previous studies have indicated that the mammalian target of rapamycin (mTOR) signaling pathway may be important in the course of epilepsy and epileptogenesis, and may offer a promising molecular target in epilepsy therapy. mTOR, a serine/threonine kinase, is a critical regulator of neuronal functions, including cellular metabolism, survival, growth, proliferation and plasticity (4). It can be activated through the phosphoinositide 3-kinase (PI3K)/Akt and Ras/mitogen-activated protein kinase kinase (MEK)/extracellular signal-regulated kinase (ERK) pathways, and inhibited by the energy sensor, 5′adenosine monophosphate(AMP)-activated protein kinase (5). mTOR exists as two complexes, mTORC1 and mTORC2. mTORC1 activates the p70 ribosomal protein S6 kinase and inactivates eIF4E binding protein 1, to promote cell growth, however, the role of mTORC2 remains to be fully elucidated (6,7).

The peroxisome proliferator-activated receptors (PPARs) constitute a class of nuclear transcription factors belonging to the nuclear receptor superfamily. There are three subtypes, α, β (or δ) and γ, among which PPAR-γ has been investigated extensively. PPAR-γ is involved in immune regulation, differentiation, glucose metabolism and the synthesis of triglycerides. It is expressed in the central nervous system and has a neuroprotective role (8). Natural ligands of PPAR-γ include the fatty acid metabolites, 9-hydroxyoctadecadienoic acid and 13-hydroxyoctadecadienoic acid, 12-hydroxy-5,8,10,14 eicosatetraenoic acid, 15-hydroxy-5,8,11,14 eicosatetraenoic acid and prostaglandins (9). Synthetic ligands of PPAR-γ include predominantly thiazolidinedione ketones. Among these, pioglitazone and rosiglitazone are used clinically, and ciglitazone and troglitazone are used experimentally. The thiazolidinedione ketones also comprise non-steroidal anti-inflammatory drugs, including diclofenac and anti-nfan (10).

The association between PPAR-γ agonists and the mTOR pathway is complex and remains to be fully elucidated. Indirect evidence suggests that PPAR-γ agonists may suppress the mTOR pathway (11), whereas other studies have suggested they may function as activators (12,13). The present study aimed to investigate the effect of the pioglitazone PPAR-γ agonist on the mTOR pathway in pentylenetetrazol (PTZ)-induced status epilepticus (SE), and examine the expression levels of the IL-1β and IL-6 inflammatory factors.

## Materials and methods

All the experimental protocols and procedures were approved by the Ethics Committee of Harbin Medical University (Harbin, China).

### SE animal model and seizure monitoring

Adult male Sprague-Dawley rats (n=126; Laboratory Animal Center of the Second Affiliated Hospital of Harbin Medical University) weighing 200–300 g were used in the present study. The rats were acclimatized in a dedicated animal room under a 12 h light/dark cycle at 22±2°C, and allowed free access to food and water.

SE was induced by repetitive intraperitoneal injections of subconvulsive doses of PTZ (10 mg/ml in saline; Sigma-Aldrich, St. Louis, MO, USA), as previously described (14–17). Briefly, a dose of 40 mg/kg was administered initially, followed by 20 mg/kg 10 min later. Subsequent doses of 10 mg/kg were administered to the rats at 10 min intervals until SE occurred (18). This procedure led to an SE lasting at least 30 min and consisting of prolonged episodes of seizures, interrupted by postictal depression phases with no return to a quadruped posture or consciousness. When SE lasted for ≥1 h, or if death appeared imminent, intraperitoneal injection of SE diazepam (4 mg/kg; Sigma-Aldrich), was administered. If a rat died during the establishment of SE, another rat was randomly selected for replacement.

### Experimental procedure and drug administration

The present study determined the time course of activation of the mTOR signaling pathway in SE. The rats were randomly distributed into either an SE group or a control group (n=21 each). In each group, three rats were sacrificed by decapitation under anesthesia with intraperitoneal sodium pentobarbital (20 mg/ml in saline; 40 mg/kg; Beijing Solarbio Science & Technology Co., Ltd., Beijing, China) at each of seven specific time points following modeling, at 1, 8, and 16 h, and on days 2, 3, 5 and 7. Western blot analyses were performed to detect the protein levels of mTOR, phosphorylated (p-)mTOR, S6 and p-S6 subsequent to acquisition of hippocampal tissues. The peak time-period of mTOR signaling pathway activation was then used in the subsequent experiments.

The effect of the pioglitazone PPAR-γ agonist on the activation of the mTOR signaling pathway in SE was then assessed. The rats were randomly assigned to four groups (11 rats/group), and received daily intragastric treatments for 5 days, as follows: Normal control group, 10 mg/kg saline (vehicle); pioglitazone-treated group, 10 mg/kg pioglitazone (Sigma-Aldrich); vehicle+SE goup, 10 mg/kg saline, with SE triggered 30 min following the final intraperitoneal injection of PTZ; and the pioglitazone+SE group, 10 mg/kg pioglitazone, with SE similarly triggered 30 min after the final intraperitoneal injection of PTZ.

All the rats were sacrificed at the time-point when mTOR signaling changes reached their peak, as determined in the first experiment. The entire hippocampal tissues were obtained, Nissl staining was performed to detect neuronal loss in the CA3 area. An enzyme-linked immunosorbent assay (ELISA) was performed to quantify the levels of IL-1β and IL-6 and immu-nohistochemical detection was performed to assess the levels of p-mTOR in the CA3 area. Western blot analysis was performed to quantify the protein levels of mTOR, p-mTOR, S6 and p-S6.

### ELISA for the detection of IL-1β and IL-6 in hippocampal tissues

The levels of IL-1β and IL-6 in the hippocampal tissues 24 h after SE were analyzed using an ELISA. The procedures were performed, according to the manufacturer’s instructions (Neobioscience Technology, Beijing, China).

The entire hippocampus was homogenized, ground and centrifuged for 30 min (4°C; 10,000 × g). The supernatant was then collected. The protein concentrations were determined using the Bradford method (19). Equal quantities of the lysates were used for the analyses of IL-1β and IL-6, with values expressed as pg/ml protein.

### Immunohistochemistry

Staining of p-mTOR (S2448) was performed, as follows: Following the embedding of the hippocampal tissues in paraffin (Shanghai Hualing Recovery Appliance Factory, Shanghai, China), sections (5 *µ*m) from the CA3 area were transferred to slides (Leica RM 2135, Leica Microsystems GmbH, Wetzlar, Germany) and deparaffinized. The slides were then incubated with citrate buffer (pH 6; Beijing Solarbio Science & Technology Co., Ltd.) for 5 min in a microwave oven twice (with a 10 min interval between) and cooled to room temperature. The slides were then incubated with rabbit p-mTOR polyclonal antibody (Ser2448; 1:65; YP0176; ImmunoWay Biotechnology Company, Newark, DE, USA) at 4°C overnight, and then in the anti-rabbit secondary immunoglobulin (Ig)G antibody conjugated to horseradish peroxidase (PV6001; OriGene Technologies, Inc., Beijing, China) for 30 min. A 3,3′-diaminobenzidine kit (ZLI-9017; OriGene Technologies, Inc.) was used for visualization, and slides were counterstained with hematoxylin (Beijing Solarbio Science & Technology Co., Ltd.). The average integrated optical density value was obtained by analyzing five fields per slide using Image-Pro Plus software (v. 6.0; Media Cybernetics, Carlsbad, CA, USA).

### Nissl staining

Nissl staining was performed, as described previously (20). Briefly, following deparaffinization in xylene (Tianjin Fuyu Fine Chemical Co., Ltd., Tianjin, China) and hydration in 100% alcohol twice for 5 min, 95% alcohol for 3 min and 70% alcohol for 3 min, the sections were stained in 0.1% cresyl violet solution (Sigma-Aldrich) for 10 min at room temperature. The slides were then rinsed rapidly in distilled water, differentiated in 95% ethyl alcohol for 2–30 min, in order to determine the optimal time of 10 min, and checked under a microscope. Slides were then dehydrated and mounted. The average integrated optical density was obtained by analyzing five fields per slide using Image-Pro Plus software (v. 6.0). The nissl bodies appeared blue-purple and cell nuclei appeared blue.

### Western blot analysis

Western blot analysis was performed in accordance with previously desctribed methods (21). The hippocampal tissues were homogenized and lysed with radioimmunoprecipitation lysis buffer (150 *µ*l/20 mg tissue; Beyotime Institute of Biotechnology, Haimen, China) and phenylmethanesulfonylfluoride (Beyotime Institute of Biotechnology). Following centrifugation at 10,000 × g for 30 min at 4°C, the supernatant was collected. Equivalent quan-tites of protein (10 *µ*l/lane) were resolved via 10% SDS-PAGE (Beyotime Institute of Biotechnology) and transferred onto a polyvinylidene fluoride membrane (EMD Millipore, Billerica, MA, USA).

Western blot analyses were performed using the following rabbit polyclonal antibodies from ImmunoWay Biotechnology Company: mTOR (1:500; YT2915), p-mTOR (Ser2448; 1:500; YP0176), S6 (1:500; YT4139), p-S6 (1:500; YP0893) and β-actin antibodies (1:1,000; YT0099) for 2 h at 37°C or overnight at 4°C. The membrane was then incubated with the secondary antibody, alkaline phosphatase-conjugated anti-rabbit IgG (1:500; ZB2308; OriGene Technologies, Inc.) and detected using Western Blue Stabilized Substrate for Alkaline Phosphatase (Promega Corporation, Madison, WI, USA). The protein levels were normalized against β-actin, and the phosphorylation of the proteins were compared with the total protein.

### Statistical analysis

Statistical analysis was performed using SPSS software, version 13.0 (SPSS, Inc., Chicago, IL, USA). Unless otherwise stated, all data were analyzed using Student’s t-test or one way analysis of variance. The data are presented as the mean ± standard deviation. P<0.05 was considered to indicate a statistically significant difference.

## Results

### Behavioral changes and activation of mTOR signaling in SE

The present study first determined whether mTOR signaling was activated in PTZ-induced SE. SE was successfully elicited in the experimental rats (n=40). At the beginning of SE, the rat typically engaged in washing of the face, nodding and rhythmic chewing for <3 min in each episode. Following this, recurrent, generalized tonic-clonic seizures, with standing, falling and tumbling became apparent. The mortality rate of the PTZ-treated rats was 47.5% (19/40 rats).

**Figure 1:**
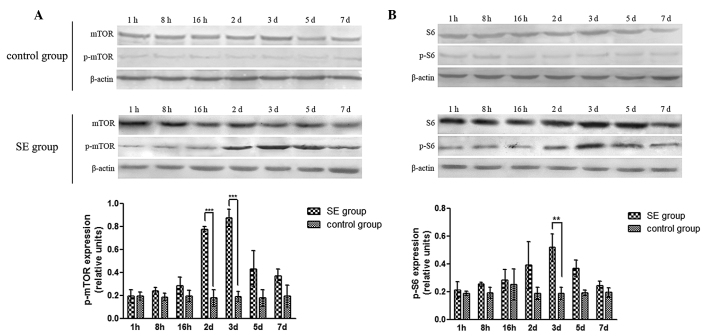
Protein expression of mTOR signaling in PTZ-induced SE. (A) Representative images and quantitative analysis of the ratio of p-mTOR/mTOR. (B) Representative images and quantitative analysis of the ratio of p-S6/S6. Data are expressed as the mean ± standard deviation.^**^P<0. 01 and ^***^P<0.001. mTOR, mammalian target of rapamycin; PTZ, pentylenetetrazol; p-, phosphorylated; SE, status epilepticus.

The levels of mTOR, p-mTOR, S6, and p-S6 were then determined using western blot analysis, and the p-mTOR/mTOR and p-S6/S6 ratios were calculated to determine the activation of mTOR signaling between 1 h and 7 days. The results demonstrated no significant changes in the expression of mTOR or S6 in the SE group (P>0.05), while the expression levels of p-mTOR and p-S6 were signifi-cantly increased on the 2nd day and peaked on the 3rd day (P<0.05; Fig. 1). No significant changes in mTOR, p-mTOR, S6, or p-S6 were observed in the control group. Similarly, the p-mTOR/mTOR and p-S6/S6 ratios in the SE group were significantly increased on the 2nd day, and peaked on the 3rd day, compared with those in the control group (P<0.05).

The results of the present study indicated that mTOR signaling was activated in PTZ-induced SE, and the most marked changes in the p-mTOR/mTOR and p-S6/S6 ratios occurred at the day 3. Therefore, in subsequent investigations, the 3rd day was selected as the point of examination.

**Figure 2:**
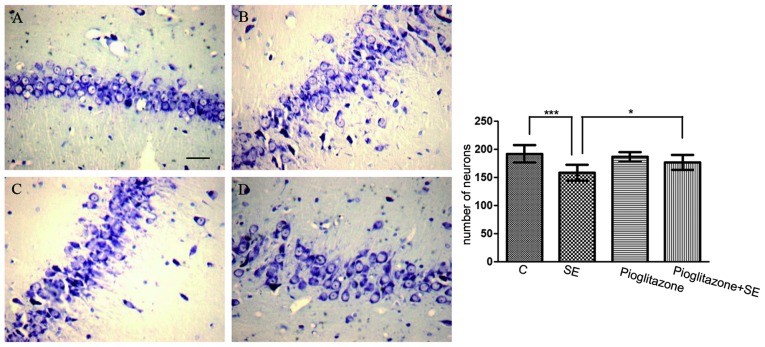
Neuronal loss in the hippocampal CA3 area following SE. Neuronal damage was assessed histologically using Nissl staining. Compared with the (A) control group, more dark colored, disorganized and blurred Nissl bodies were found in the (B) vehicle-treated SE group on the third day following SE, (C) slightly blurred Nissl bodies were observed in the pioglitazone group and the number of Nissl bodies decreased in the (D) pioglitazone-treated SE group. Scale bar=20 *µ*m. Data are expressed as the mean ± standard deviation. ^*^P<0.05 and ^***^P<0.001. C, control; SE, status epilepticus.

In addition, neuronal loss was detected subsequent to SE. The number of surviving neurons in the CA3 area in the vehicle treated-SE group was significantly reduced compared with that in the control group (P<0.001; Fig. 2A and B).

### Effects of pioglitazone on neuroprotection, activation of the mTOR pathway and the levels of IL-1β/IL-6 in SE

The effect of the pioglitazone PPAR-γ agonist on the SE-induced changes was also examined. The mean duration required to trigger SE was 31.30±6.22 min in the vehicle-treated SE group, compared with 44.70±14.27 min in the pioglitazone-treated SE group (P<0.05). In addition, the number of surviving neurons decreased in the SE group, while the number of surviving neurons in the pioglitazone-treated SE group was higher, compared with the number in the vehicle treated-SE group (P<0.05; Fig. 2). These data indicated that pioglitazone had a neuroprotective role in SE.

**Figure 3:**
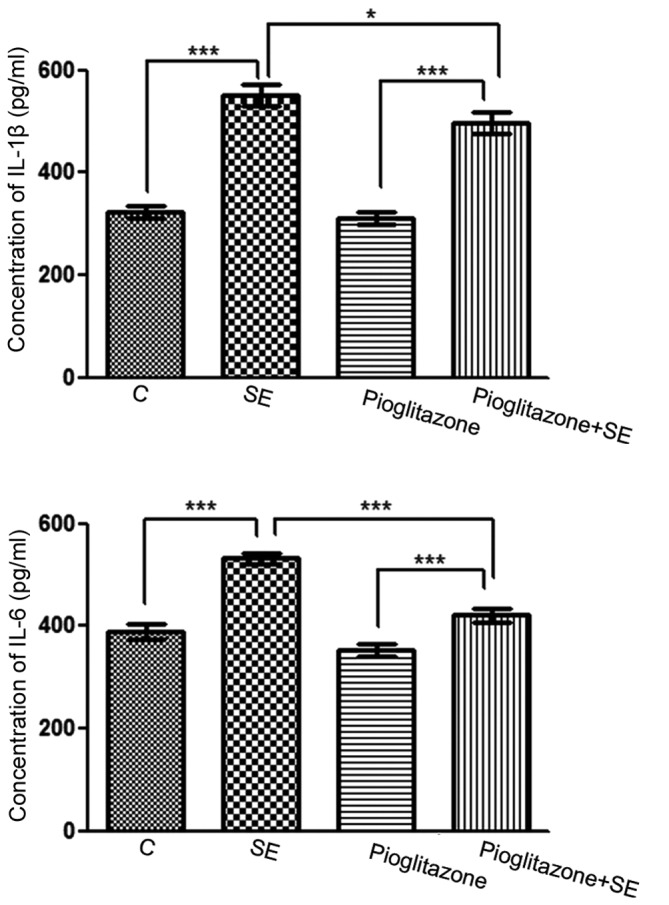
Effects of pioglitazone on hippocampal levls of IL-1β/IL-6, detected using an enzyme-linked immunosorbent assay. Equal quantities of lysates were used for IL-1β and IL-6 analysis. Values are expressed in pg/ml protein and are presented as the mean ± standard deviation. ^*^P<0. 05 and ^***^P<0.001. C, control; SE, status epilepticus; IL, interleukin.

To assess whether pioglitazone was associated with lower levels of proinflammatory cytokines, the levels of IL-1β and IL-6 in the hippocampal tissues were examined using ELISA. The results revealed that the levels of IL-1β and IL-6 in the vehicle-treated SE group were significantly increased compared with the control group (P<0.001). The levels of IL-1β and IL-6 in the pioglitazone-treated SE group were increased compared with the pioglitazone group (P<0.001), however, the levels of IL-1β (P<0.5) and IL-6 (P<0.001) in the pioglitazone-treated SE group were significantly decreased compared with the vehicle-treated SE group (Fig. 3). These results suggested that proinflammatory cytokines were involved in SE, and that the PPAR-γ agonist inhibited inflammation.

**Figure 4:**
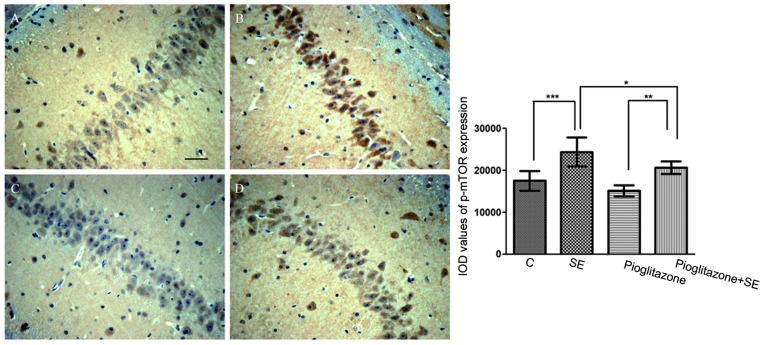
Immunohistochemical detection of the expression of p-mTOR in the CA3 area. (A) Control group, (B) vehicle-treated SE group, (C) pioglitazone group, (D) pioglitazone-treated SE group. Scale bar=20 *µ*m. Data are expressed as the mean ± standard deviation. ^*^P<0.05, ^**^P<0. 01 and ^***^P<0.001. C, control; SE, status epilepticus. p-mTOR, phosphorylated mammalian target of rapamycin.

The activation of the mTOR pathway was also detected by immunohistological analysis (Fig. 4). The cells positive for p-mTOR in the vehicle treated-SE group were significantly increased compared with those in the control group (P<0.001).

**Figure 5:**
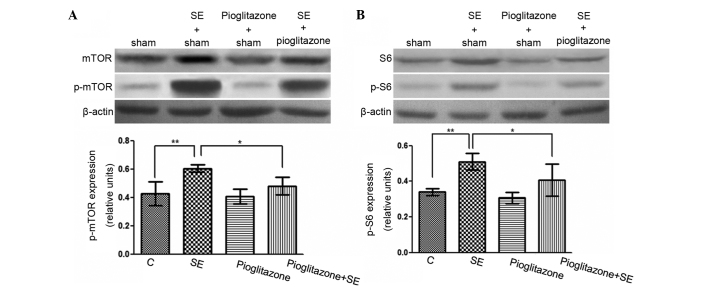
Effects of pioglitazone on the protein expression of mTOR signaling in the hippocampus of SE rats. (A) Representative images and quantitative analysis of the ratio of p-mTOR/mTOR. (B) Representative images and quantitative analysis of the ratio of p-S6/S6. Data are expressed as the mean ± standard deviation. ^*^P<0.05 and ^**^P<0.01. C, control; SE, status epilepticus; p-mTOR, phosphorylated mammalian target of rapamycin.

The present study also examined whether pioglitazone had an effect on the mTOR pathway (Fig. 4). Although the number of cells positive for p-mTOR in the pioglitazone-treated SE group were higher compared with those in the pioglitazone group (P<0.01), those in the pioglitazone-treated SE group were lower compared with those in the vehicle-treated SE group (P<0.05). Similar results were detected using western blot analysis (Fig. 5). The p-mTOR/mTOR and p-S6/S6 ratios in the vehicle-treated SE group were significantly higher compared with the ratios in the control group (P<0.01), while the ratios in the pioglitazone-treated SE group were significantly lower compared with the vehicle-treated SE group (P<0.05). These data suggest that the pioglitazone PPAR-γ agonist inhibited the mTOR pathway.

## Discussion

Epileptogenesis occurs in the period between the start of brain damage, due to cerebral trauma, infection or genetic deficiency, and the first spontaneous seizure (22–24). SE is a more severe form of epilepsy compared with other forms, and is usually accompanied by damage to the brain. There is evidence suggesting that spontaneous recurrent seizures may occur in the days or weeks following SE recovery (25). In the present study, the time-points at which measurements were obtained fell within this interval of epileptogenesis.

The hippocampus has a more important role in epilepsy compared with the amygdala or cerebral cortex due to its plasticity and adaptability (26). The general structure of the hippocampus can be divided into the hippocampal cortex and the dentate gyrus. The hippocampal cortex can be further divided into the CA1, CA2, CA3 and CA4 areas (27). The CA1 and CA3 areas are particularly vulnerable to injury, as these areas lack blood vessels and are more susceptible to hypoxic-ischemic damage. In addition, N-methyl-D-aspartate (NMDA) receptors are more abundant in the CA3 area, resulting in increased vulnerability to excitatory amino acids. Therefore, the CA3 area was selected for examination in the present study.

Several drugs have been used to induce epilepsy in rats, including pilocarpine, kainate and PTZ (28). PTZ can convert inactivated K^+^ channels to open channels, decrease the action potential threshold and increase neuronal excitation, making PTZ an effective epileptic agent, which is frequently used to establish epilepsy models (29–35). In addition, intraperitoneal injection of PTZ does not disturb the structure of brain tissue, and SE can be successfully induced (36), which is the reason for the use of PTZ to induce SE in the present study.

Several studies have indicated that mTOR signaling is associated with epileptogenesis (37–39). Zeng *et al* (40) observed biphasic activation of the mTOR pathway in a kainate-induced SE model, with one peak within hours and another peak within days. It was concluded that the first peak, found in the hippocampus and cortex, may have been caused by activation of glutamate receptors, while the second peak may have occurred due to epileptogenesis, observed only in the hippocampus. Zhang *et al* (41) found that acute seizures, triggered by a single large dose of PTZ (75 mg/kg), induced the transient activation of mTOR in the hippocampus and cortex, which lasted 6 h and returned to baseline after 16 h.

In the present study, the mTOR signaling pathway was activated in the hippocampus of PTZ-induced SE rat models. However, unlike the results of Zeng *et al* (40), only a single peak of mTOR activation and downstream S6 activation was observed, on the third day. This may have been the result of the epileptogenic mechanisms of PTZ and kainatediffer. PTZ-induced seizures involve blocking of the Cl-related type A γ-aminobutyric acid receptor and NMDA receptor-mediated transmission (42), while kainate is an analogue of glutamate, an excitatory amino acid neurotransmitter in the brain (43). In addition, a second peak may have occur, beyond the observation period of the present study. PPAR-γ is an important transcription factor in adipogenesis. The results in the present study demonstrated that the pioglitazone PPAR-γ agonist inhibited the mTOR signaling pathway, which was detected by western blot and immunochemical analyses. A previous study (9) demonstrated that PPAR-γ agonists increase the expression of PTEN, a phosphatase and tensin homolog, in adipocytes and skeletal muscle cells. PTEN is a negative regulator of mTOR, therefore, a PPAR-γ agonist may repress the mTOR pathway. Another study suggested that mTOR inhibitors can inhibit the positive feedback loop of transcription between CCAAT-enhancer-binding protein-α and PPAR-γ to suppress their expressio, thereby suppressing adipogenesis and adipocyte differentiation (44), and indicating that the inhibition of the mTOR pathway inhibits the expression of PPAR-γ. This appears to contradict the results of the present study, and a negative feedback loop may have been present.

There are multiple feedback loops in the mTOR pathway. For example, the mTORC1-Akt feedback loop has been demonstrated in a variety of cancer cells and xenograft tissues; rapamycin may elevate levels of p-Akt which is upstream of mTOR, but it also inhibits mTORC1 (45,46). In addition, an mTORC1-MAPK/ERK feedback loop has been reported (47,48). MAPK/ERK can positively regulate the activity of mTORC1, and elevated levels of ERK phosphorylation, induced by rapamycin, have been observed in *in vitro* cell culture, in a murine tumor model and in patients with cancer (47,48). The presence of these feedback loops may act to offset the inhibitory effect of drugs on the mTOR pathway and cause resistance. The existence of such loops requires further investigation.

Inflammation is important in epilepsy. A previous study (49) demonstrated that levels of IL-1β, IL-6 and tumor necrosis factor-α were significantly elevated in the seizure focus. It also reported that the mTOR pathway is involved in the activation of microglia, and that phosphatidic acids can activate the mTOR pathway (50). The activation of mTOR can also promote the release of the IL-10 inflammatory factor (51), whereas inhibition of mTOR can reduce the activity of macrophages and microglial cells, thereby reducing nerve inflammation (52).

The neuroprotective role of PPAR-γ agonists has been previously observed in models of central nervous system diseases, including acute cerebral ischemia, Parkinson’s disease and Alzheimer’s disease (53–55). In the present study, the pre-administration of pioglitazone reduced neuronal loss following SE. The results also demonstrated that, in SE, the pioglitazone PPAR-γ agonist inhibited the mTOR pathway, and decreased the levels of IL-1β and IL-6 in the hippocampus. These results suggested that a PPAR-γ agonist may assist in relieving SE by inhibiting the mTOR pathway and by decreasing inflammation factors. Further experiments are required to investigate the associations among the PPAR-γ agonists, mTOR pathway and anti-inflammatory effects.

Notably, the present study demonstrated that neuronal loss in the pioglitazone-treated group was greater compared with that in the control group, although the difference was not statistically significant. It was suggested that this was due to hypoglycemia following pioglitazone treatment; however, the rats were found to be normoglycemic following pioglitazone administration. Therefore, there may be additional mechanisms or pioglitazone may act as a double-edged sword, which requires further investigation.

In conclusion, the results of the presents study suggested that the mTOR pathway was activated in PTZ-induced SE rats, and its activation peaked on the third day. In addition, the PPAR-γ agonist was associated with anti-inflammatory effects and inhibition of the mTOR pathway. Further investigations are required to examine the associations among PPAR-γ agonists, the mTOR pathway, and inflammatory factors, and to delineate a PPAR-γ-mTOR feedback loop.

## Abbreviations

4E-BP1: eukaryotic translation initiation factor 4E-binding protein 1
9-13-HODE: 9-hydroxyoctadecadienoic acid and 13-hydroxyoctadecadienoic acid
12-HETE: 12-hydroxy- 5,8,10,14 eicosatetraenoic acid
15-HETE: 15-hydroxy-5,8,11,14 eicosatetraenoic acid
AMPK: 5′adenosine monophosphate-activated protein kinase
mTOR: mammalian target of rapamycin
PPAR: peroxisome proliferator-activated receptor
PPAR-γ: peroxisome proliferator-activated receptor γ
PTZ: pentylenetetrazol
S6K1: p70 ribosomal protein S6 kinase
SE: status epilepticus
